# Biotinylated Quinone as a Chemiluminescence Sensor for Biotin-Avidin Interaction and Biotin Detection Application

**DOI:** 10.3390/s23239611

**Published:** 2023-12-04

**Authors:** Fatema Kaladari, Mahmoud El-Maghrabey, Megumi Kawazato, Naoya Kishikawa, Naotaka Kuroda

**Affiliations:** 1 Graduate School of Biomedical Sciences, Course of Pharmaceutical Sciences, Nagasaki University, 1-14 Bunkyo-machi, Nagasaki 852-8521, Japan; bb55721951@ms.nagasaki-u.ac.jp (F.K.); mh-elmaghrabey@nagasaki-u.ac.jp (M.E.-M.); bb55622008@ms.nagasaki-u.ac.jp (M.K.); kishika@nagasaki-u.ac.jp (N.K.); 2 Pharmaceutical Analytical Chemistry Department, Faculty of Pharmacy, Mansoura University, Mansoura 35516, Egypt

**Keywords:** biotin, avidin, biotin-naphthoquinone, proximity assay, chemiluminescence, supplement tablets

## Abstract

Biotin, or vitamin B7, is essential for metabolic reactions. It must be obtained from external sources such as food and biotin/vitamin supplements because it is not biosynthesized by mammals. Therefore, there is a need to monitor its levels in supplements. However, biotin detection methods, which include chromatographic, immune, enzymatic, and microbial assays, are tedious, time-consuming, and expensive. Thus, we synthesized a product called biotin-naphthoquinone, which produces chemiluminescence upon its redox cycle reaction with dithiothreitol and luminol; then it was used as a chemiluminescence sensor for biotin–avidin interaction. When a quinone biotinylated compound binds avidin, the chemiluminescence decreases noticeably due to the proximity between quinone and avidin, and when free biotin is added in a competitive assay, the chemiluminescence returns. The chemiluminescence is regained as the free biotin displaces biotinylated quinone in its complex with avidin, freeing biotin-naphthoquinone. Many experiments, including the use of a biotin-free quinone, proved the competitive nature of the assay. The competitive assay method used in this study was linear in the range of 1.0–100 µM with a detection limit of 0.58 µM. The competitive chemiluminescence assay could detect biotin in vitamin B7 tablets with good recovery of 91.3 to 110% and respectable precision (RSD < 8.7%).

## 1. Introduction

Biotin is a water-soluble compound belonging to the vitamin B group and is also known as vitamin B7. Biotin is covalently attached to the active sites of key metabolic enzymes such as biotin carboxylase and decarboxylase [1]. Catalyzing carboxyl group transfers to organic acids is one of the most important functions of these enzymes. Additionally, biotin acts as a carboxyl carrier from one compound to the other. Biotin can be found in free and protein-bound (Biocytin) forms in nature [2].

Plants, bacteria, and fungi are the only organisms capable of synthesizing biotin, and thus, mammals must obtain it from external sources. Biotin can be obtained from natural sources such as fruits and vegetables in free form and in protein-binding form from meat and grain [3]. However, natural foods usually have a small amount of biotin, especially vegetables, and the amount is somewhat higher in eggs and liver; nevertheless, it is still not enough [2]. Depending on the age group, different amounts of biotin are required daily. For infants, the recommended intake ranges from 4 to 6 µg per day [4], and for children, it is 20 µg per day [5]. Finally, for adults, the recommended intake is 40–45 µg per day [6]. Biotin deficiency can lead to several diseases, such as red skin rash, depression, hallucination, hypotonia in infants, dermatitis, seizures, developmental delay, hearing loss, and alopecia [3,7,8,9]. In the case of pregnancy, a deficiency in biotin causes various malformations of the fetus and could lead to fetal mortality [2]. Early detection of biotin deficiency, followed by supplementation, is critical for its treatment. Furthermore, biotin supplementation has proven to have various benefits, such as improving hair loss and skin abnormalities, such as dermatitis, alongside contributing to the control of irritation in acne [10]. Therefore, there is a need to monitor its levels in foods and supplements [11].

There are several methods used to determine biotin levels in foods, pharmaceuticals, and biofluids, such as agar plates [12], HPLC-UV [13], liquid chromatography-mass spectrometry (LC-MS) [2], and HPLC-FL post-column binding assays [14]. The microbiological method depends on the presence of biotin as a necessary nutrient for the cultivation of microorganisms. For biotin microbiological determination, certain microorganism cultures are cultivated with biotin standards and samples. Afterward, the culture development is quantified, for example, using the growth diameter in the plate [15]. The procedure depends on treating the samples with acid (H_2_SO_4_) to release free biotin, followed by neutralization via NaOH and addition to wells in the agar plate. The most common biotin-dependent microorganism is *Lactobacillus plantarum* [16]. In a study with a great recovery of biotin concentrations in human serum and urine, Fukui et al. developed an improved version of the agar plate method using *Lactobacillus plantarum*, and they stained the bacterial growth with bromocresol purple [12].

Ekpe et al. determined biotin in multivitamin-multimineral tablets by using reverse-phase high-pressure liquid chromatography (RP-HPLC). The method separated biotin using an octyl silane column, and the UV detection was performed at 200 nm wavelength, showing good precision and recovery [13]. Additionally, Thompson et al. reported using HPLC with a reversed-phase C18 column with a conjugation reaction with streptavidin-fluorescein isothiocyanate. This method used a fluorescence detector with a short chromatography run time to detect biotin in infant formula, medical and nutritional products, and vitamin premix samples [14]. Plinton et al. developed a colorimetric method to detect biotin in premixes with dicalcium phosphate. A potassium iodate solution and an acidic solution were used to oxidize biotin to sulfone and convert iodate to iodine. In this method, the absorbance of iodine produced at 520 nm is proportional to the concentration of biotin [17]. Lastly, Luu Thi Huyen et al. developed a method using liquid chromatography-tandem mass spectrometry (LC-MS/MS) to determine biotin levels in nutritional products and supplements. The method had fast and simple pretreatment and good recovery [2].

These previously reported methods for determining biotin levels in foods and supplements could be regarded as reliable, but they were also complex, time-consuming, and expensive, and they lacked selectivity. For instance, the conventional method of using a UV detector lacks sensitivity because biotin has no chromophores and has only a weak absorbance at wavelengths of 200–210 nm, which overlaps with the wavelength of many other solvents. Moreover, the microbiological method displays different selectivity for biotin and its derivatives. Although HPLC-based methods have higher sensitivity than others, they require pretreatment of the sample, are tedious, and could be of lower sensitivity when coupled with UV detection. The use of an MS detector after chromatographic separation rendered the analysis very sensitive and selective; however, it is expensive and requires an experienced analyst. Also, the colorimetric method developed by Plinton et al. is nonspecific, and the sample medium can easily interfere with the reaction; hence, it requires separation steps [17]. Overall, all methods to detect biotin can give reliable results regarding its level in different matrices; however, they sometimes lack sensitivity and selectivity and require several tiresome steps, including complicated sample pretreatment.

**Scheme 1:**
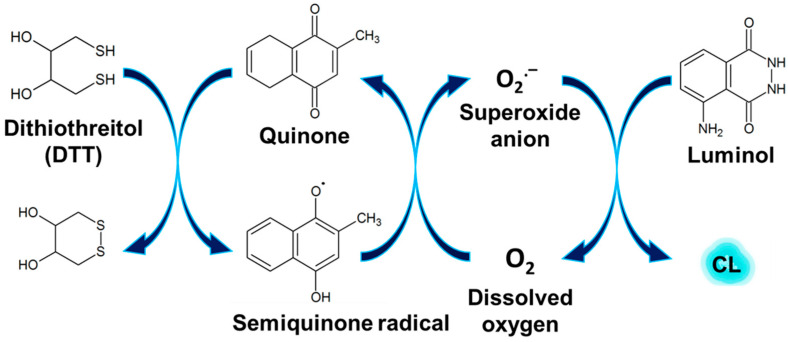
Redox cycle of quinone upon its reaction with the reductant DTT and luminol. CL hear stands for chemiluminescence.

In our laboratory, we have developed selective chemiluminescence (CL) analytical techniques for quinone. Quinone can act as an initiator of reactive oxygen species (ROS). The developed selective CL analytical techniques for quinones utilize the generation of ROS in a redox cycle reaction, as shown in Scheme 1 [18,19]. Quinone reacts with a reductant such as a dithiothreitol (DTT) and produces a superoxide anion radical, which reacts with luminol to produce a CL signal in the redox cycle. This reaction was used by our research group for the analysis of many pharmaceutical quinones, including, but not limited to, pyrroloquinoline quinone [18] and doxorubicin [19].

Additionally, quinones were used as a new alternative for replacing enzymes in enzyme-linked immunosorbent assay (ELISA), as our research groups have developed quinone-linked immunosorbent assay (QuLISA) [20] and multi-QuLISA [21,22].

One product that was synthesized in our laboratory is called biotin-1,2-naphthoquinone (BT-NQ); this can undergo the redox cycle with high sensitivity [20]. BT-NQ was used in immunoassay via the avidin system, and we discovered that in the presence of avidin, the CL intensity (CLI) decreases noticeably. Moreover, in the NQ-avidin system with no biotin to act as a bond, the CL was unfazed, but in the BT-NQ-avidin system, the CL decreased due to the proximity between quinone and avidin. Upon addition of free biotin, the CL was regained in a concentration-dependent manner related to free biotin. This is due to the competition of free biotin with BT-NQ for the avidin sites, and upon binding of biotin with avidin, BT-NQ is released and regains its CL. Thus, we developed a competitive assay that depends on the proximity between BT-NQ and avidin for the detection of biotin in tablets.

## 2. Materials and Methods

### 2.1. Chemicals and Solutions

Avidin was purchased from Calzyme Laboratories, Inc. (San Luis Obispo, CA, USA); 1,2-Naphthoquinone-4-sulfonate (NQS) and biotin were from Sigma-Aldrich (St. Louis, MO, USA). Luminol, DTT, and dimethylformamide (DMF) were obtained from Nacalai Tesque (Kyoto, Japan). Biotin hydrazide and sodium hydroxide were bought from Sigma-Aldrich (St. Louis, MO, USA). Biotin supplement (Brand DHC) was purchased from a local drug store in Nagasaki, Japan. BT-NQ solution was prepared in a 5% DMF aqueous solution. Luminol solution was prepared in 0.1 M NaOH. The DTT, avidin, and biotin solutions were prepared in deionized water.

### 2.2. Instruments

A Lumat LB-9507 luminometer (Berthold Technologies, Bad Wildbad, Germany) was used for all the CL-related studies and assays with a measurement time interval of 1 s, total integration time of 300 s, and measurement wavelength range from 390 to 620 nm. A Varian5-inova500 (500 MHz) spectrometer (Lake Forest, CA, USA) was used to measure the BT-NQ ^1^H-NMR spectrum in DMSO-d6. A JEOL JMS700N spectrometer (Tokyo, Japan) was used for the measurement of the fast atom bombardment-mass spectrometry (FAB-MS) spectrum of BT-NQ using chloroform, DMF, and glycerol as matrices.

### 2.3. Synthesis of Biotin-1,2-Naphthoquinone (BT-NQ)

BT-NQ was prepared using a method similar to that described in our previous report [20] but with small modifications. In brief, 50 mg of NQS was dissolved in 8 mL of hot water, and 50 mg of biotin hydrazide was dissolved in 8 mL of ethanol: water (1:1) mixture. After two hours in the sonicator, the solutions were mixed in a flask with heat and stirring reflux for 5 h. The reaction mixture was then filtrated, and the solid was washed with hot water to remove excess substrate from the surface of the reaction product. The mass obtained was 49 mg of orange crystals with a yield of 61.6%.

### 2.4. Calibration Curve of Free Standard Biotin in Competitive Assay

An aliquot of 200 µL of avidin (30 µM) was added into the Eppendorf tube, followed by the addition of 100 µL of different concentrations (5–100 µM) of biotin and 30 µM of BT-NQ. The mixture was incubated for 30 min at 37 °C, and then 100 µL was taken from the mixture and added to a test tube. Next, 50 µL of 190 µM luminol was added to the test tube and vortexed for 5 s, followed by the addition of 50 µL of 50 µM DTT. Lastly, the CLI was measured for 300 s. The intra-day and interlay precision of the method was studied by analyzing different concentrations of biotin three times on the same day and on three different days subsequently, and the relative standard deviation was calculated every time.

### 2.5. Detection of Biotin in Supplements

First, five tablets were ground and dissolved in 100 mL of water (total biotin concentration was 100 µM), then sonicated for 1 h and filtered by vacuum. Then, the solution was filtered again by a syringe filter (0.45 µM) and diluted to different concentrations. An Aliquot of 300 µL of avidin (30 µM) was added into the Eppendorf tube, followed by the addition of 150 µL of different concentrations (0–100 µM) of tablet solution and 30 µM of BT-NQ. The mixture was incubated for 30 min at 37 °C, and then 100 µL was taken from the mixture and added to a test tube. Next, 50 µL of 190 µM luminol was added to the test tube and vortexed for 5 s, followed by the addition of 50 µL of 50 µM DTT. Lastly, the CLI was measured for 300 s.

## 3. Results and Discussions

### 3.1. BT-NQ Synthesis and Characterization

**Scheme 2:**
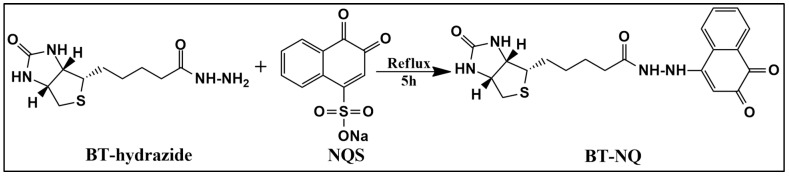
The reaction scheme used for the synthesis of BT-NQ.

BT-NQ was synthesized, as reported previously by our research group [20], using a simple reaction between biotin hydrazide and NQS, as illustrated in Scheme 2.

**Figure 1:**
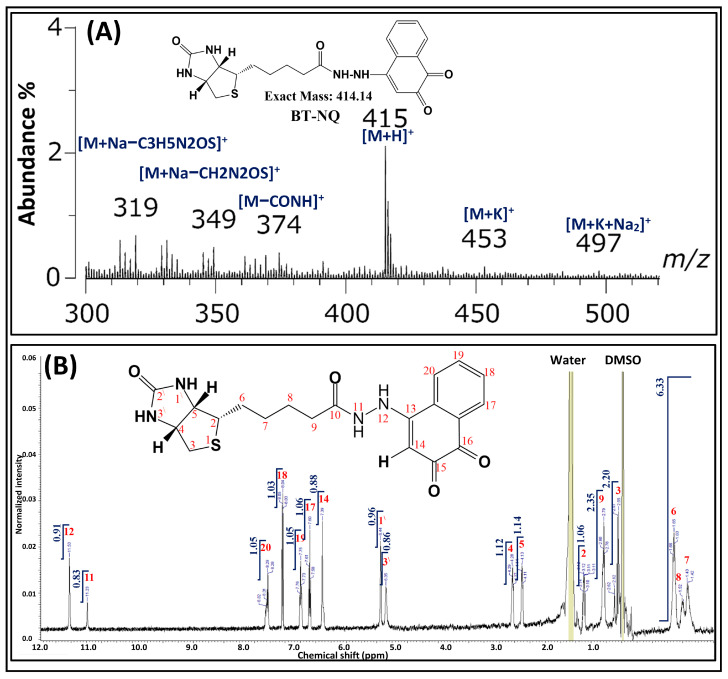
Characterization of the synthesized BT-NQ, where (**A**) FAB-MS spectrum of BT-NQ and (**B**) BT-NQ ^1^H-NMR spectrum.

BT-NQ consists of one biotin and one naphthoquinone molecule with a molecular weight of 414.43 g/mole. The structure characterization was reconfirmed via mass spectroscopy, as shown in Figure 1A. FAB-MS analysis of BT-NQ showed a molecular ion peak at 415, which corresponds to [M+H]^+^. Other fragment ions at 453 and 374 correspond to [M+K]^+^ and [M+H−CO-NH]^+^, respectively.

Next, to confirm the presence of biotin moiety in the reaction product, BT-NQ was mixed with 4-dimethylaminocinnamaldehyde, which yielded a red color, confirming the presence of biotin in BT-NQ. Afterward, the ^1^H-NMR spectra of BT-NQ (Figure 1B) were measured, and this showed the following chemical shifts (δ, ppm): δ 1.4 (2 H, CH_2_, m), δ 1.5 (2 H, CH_2_, m), δ 1.6 (2 H, CH_2_, m), δ 2.6 (2 H, S-CH_2_, m), δ 2.8 (2 H, COCH_2_, t), δ 3.1 (1 H, S-CH, m), δ 4.1 (1 H, CH, m), δ 4.3 (1 H, CH, m), δ 6.3 (1 H, NH, S), δ 6.4 (1 H, NH, S), δ 7.4 (1 H, Ar-H, S), δ 7.6 (1 H, Ar-H, m), δ 7.8 (1 H, Ar-H, m), δ 8.0 (1 H, Ar-H, m), δ 8.3 (1 H, Ar-H, m), δ 11.2 (1 H, NH, S), δ 11.5 (1 H, NH, S). The results of ^1^H-NMR and FAB-MS confirmatory tests approved the successful synthesis of biotin-naphthoquinone.

### 3.2. Interaction of BT-NQ with Avidin

**Scheme 3:**
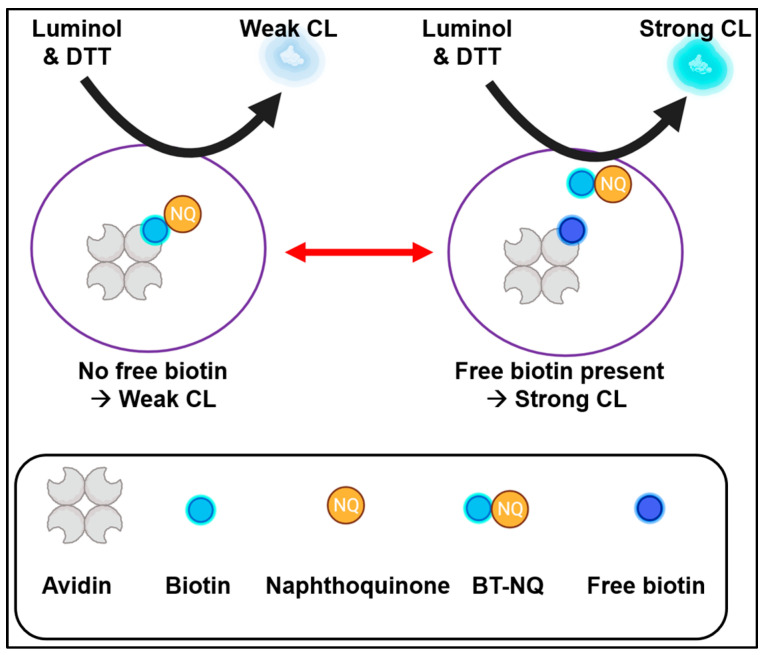
The concept of competitive CL assay between free biotin and BT-NQ based on proximity. Created with Biorender.

**Figure 2:**
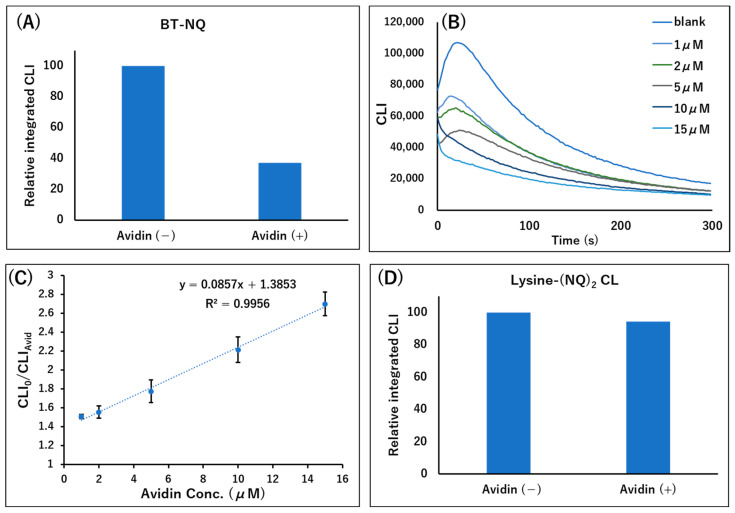
The quenching effect of avidin on the CL of BT-NQ, where (**A**) shows the effect of 15 μM avidin on the CL of BT-NQ, (**B**) the CL time spectra of BT-NQ in presence of different avidin concentrations (0–15.0 μM), and (**C**) the calibration curve for avidin concentrations (1–15 μM) vs. CLI_0_/CLI_Avid_; CLI_0_ is the CLI of BT-NQ alone, and CLI_Avid_ is the CLI of BT-NQ in presence of different concentrations of avidin. On the other hand, (**D**) shows the CL of lysine-(NQ)_2_ in the presence and absence of avidin.

First, the interaction between BT-NQ and avidin shown in Scheme 3 was studied. In the absence and presence of avidin, it was discovered that the CLI decreased due to the biotin in BT-NQ attaching to the avidin, as illustrated in Figure 2A. The quenching effect of avidin on the CL of BT-NQ is concentration-dependent, as shown in the CL time spectra of BT-NQ in the presence of different avidin concentrations (Figure 2B). A calibration graph for avidin concentrations (1–15 μm) vs. CLI_0_/CLI_Avid_ was constructed. CLI_0_ is the CLI of BT-NQ alone, and CLI_Avid_ is the CLI of BT-NQ in the presence of different concentrations of avidin. This graph showed very good linearity with a coefficient of determination exceeding 0.99 (Figure 2C). This is due to the steric hindrance caused by avidin, which caused a quenching effect on the redox cycle shown in Scheme 1.

To confirm the hypothesis that the quenching is due to the proximity between NQ and avidin through biotin–avidin complex formation, a biotin-free quinone, namely lysine-(NQ)_2_, which is a dimer consisting of a lysine molecule and two naphthoquinones, was synthesized according to the method described in a previous report [21]. The CLI of lysine-(NQ)_2_, upon redox cycle with DTT and luminol, in the presence and absence of avidin, shows no difference in CLI (Figure 2D), which demonstrates that the presence of biotin attached to quinone is a necessity for this CL-quenching interaction between BT-NQ and avidin.

**Figure 3:**
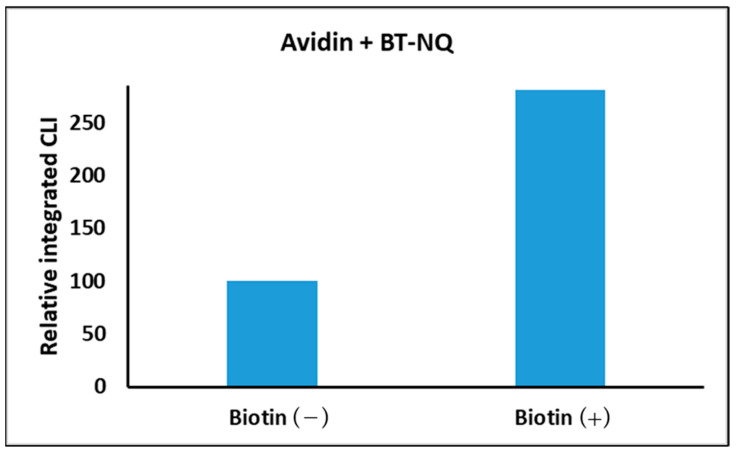
The effect of pristine biotin (100 μm) on the relative CLI of a mixture of avidin (30 µM) and BT-NQ (30 µM).

This means that proximity to avidin is the only factor affecting the CL change of BT-NQ; hence, if BT-NQ is near avidin, the CL decreases, whereas if there is free biotin competitively binding with avidin instead of BT-NQ, which was tested as shown in Figure 3, the CL will increase due to the distance between BT-NQ and avidin caused by replacing BT-NQ in the avidin complex with the free biotin (Scheme 3). This is due to the fact that pristine biotin is reported to have more affinity to avidin than modified biotin [23]. Additionally, the decrease in the CLI might be due to the steric hindrance caused by the large molecular weight of avidin, which interferes with the redox cycle shown in Scheme 1.

### 3.3. Competitive Assay of Biotin Using BT-NQ and Avidin

BT-NQ and avidin strongly interact owing to the strong association complex formed between the biotin molecule and avidin, which forms a complex with an association constant of about 10^15^ M^−1^ [23]. In a solution containing biotin, BT-NQ, and avidin, there will be a competition between the free biotin and BT-NQ for the interaction with avidin. This competition was used here to make a competitive assay for biotin determination similar to the HABA reagent assay (4′-hydroxyazobeneze-2-carboxylic acid), which is essentially a displacement test [21,22,24]. At first, the free biotin displaces the BT-NQ from its interaction with avidin because pristine biotin (in this case, the free biotin) has a higher affinity to avidin than the modified biotin (BT-NQ). Then, the second step in the mechanism is that once BT-NQ becomes displaced from avidin, it moves further away from the avidin and hence can undergo the redox cycle with DTT and luminol to produce stronger CL.

From the results in Figure 3, it is clear that biotin can free BT-NQ from avidin, which makes BT-NQ regain its redox cycle ability to produce CL upon reaction with DTT and luminol. Hence, we aimed to develop a new CL competitive assay for biotin using avidin and BT-NQ. The experiment conditions were optimized before conducting the competitive assay of BT-NQ.

**Figure 4:**
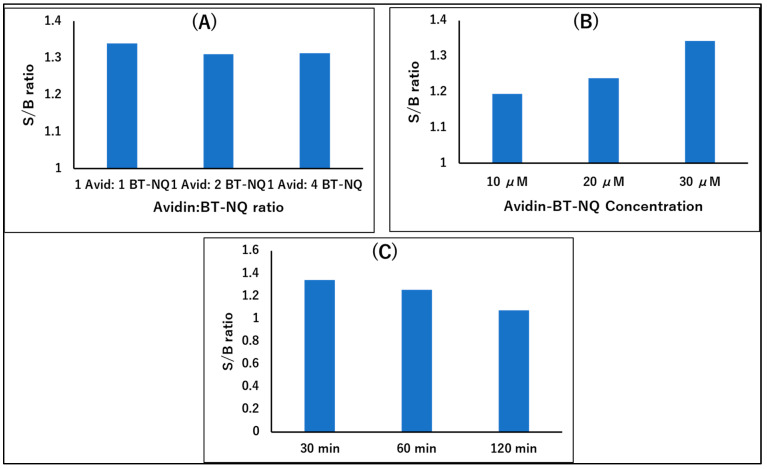
Optimization of the competitive CL assay conditions. The figure shows the effect of (**A**) Avidin:BT-NQ ratio, (**B**) Avidin-BT-NQ concentration, and (**C**) incubation time on the S/B ratio of the avidin-BT-NQ system in the presence and absence of biotin (20 µM).

During the optimization study, a biotin standard solution was used at a concentration of 20 µM. At first, different ratios of avidin and BT-NQ of 1:1, 1:2, and 1:4 were investigated, and the signal-to-blank (S/B) ratio was calculated every time. The results are shown in Figure 4A, where the best ratio that yielded the highest S/B was 1:1. Next, avidin and BT-NQ concentrations were investigated at 10, 20, and 30 µM, with the free biotin concentration kept constant at 20 µM. The optimum concentration was shown to be 30 µM (Figure 4B). Afterward, the incubation time for avidin with BT-NQ and free biotin was studied, as shown in Figure 4C. The incubation times studied were 30 min, 1 h, and 2 h. As illustrated in Figure 4C, the CLI S/B ratio decreased as the incubation time increased in the presence and absence of free biotin. At 30 min, it is clear that in the presence of free biotin, the S/B ratio is the highest; next, at 1 h incubation time, the CLI S/B decreased, and then it continued to decrease upon increasing the incubation time to 2 h. Hence, 30 min was shown to be the optimum incubation time.

**Scheme 4:**
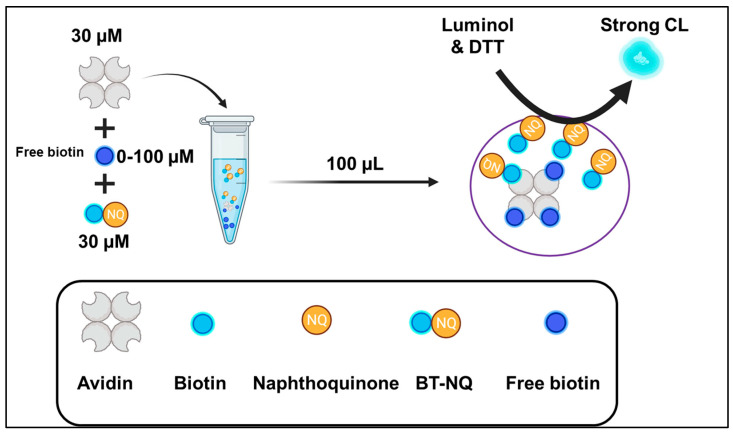
Illustration of the competitive assay between BT-NQ and free biotin. Created with Biorender.

After the optimization, the competitive assay between BT-NQ and free biotin was conducted, as clarified in Scheme 4. The competitive assay is between BT-NQ and free biotin competing for binding to avidin; as the number of free biotins increases, the more free biotin binds to the avidin, which makes more unbounded BT-NQ compounds away from avidin and thus increases the CLI (Scheme 4).

**Figure 5:**
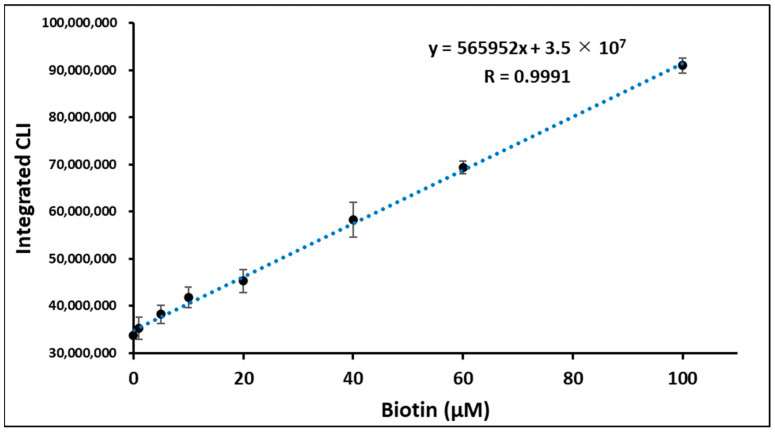
Calibration curve for biotin using avidin-BT-NQ CL system.

A calibration curve was plotted from different concentrations of free biotin ranging from 1.0 to 100.0 µM, and a linear relationship was found between the free biotin concentration and the CLI, as shown in Figure 5. The linear regression of the straight line was found to be Y = 5.66 × 10^5^ X + 3.49 × 10^7^ with a correlation coefficient (*R*) of 0.9991, where Y is the integrated CLI for 300 s, and X is the concentration of free biotin in µM. The limit of detection was found to be 0.58 µM, defined as 3δ/slope, where δ is the standard deviation of blank. The accuracy and precision (intra- and inter-day) of the developed method were studied at different concentrations of biotin. Different concentrations of standard biotin were analyzed via the proposed method, and the found % (accuracy) was calculated for every concentration using the calibration curve regression equation. Next, different concentrations were analyzed three times on the same day (intraday precision) and on three consecutive days (inter-day precision), and RSD% was calculated as an indicator of method precision. The data are summarized in Table 1. The method showed very good accuracy, with found% ranging from 98.0 to 102%, good intra-day precision of RSD% ≤ 4.45%, and acceptable intraday precision with RSD% ≤ 10.6%.

### 3.4. Application of the Competitive Assay for Determination of Biotin in Its Supplement Tablet

In the application using a biotin tablet for the competitive assay, five tablets were ground, as explained in the procedure, and after filtration, the supplement solution was diluted to different concentrations ranging from 0 to 100 µM.

Even though the supplement contained excipients such as powdered sugar, cellulose, hydroxypropyl methylcellulose, and calcium stearate, the recovery ratio was excellent, ranging from 91.3 to 110.4% with excellent precision showing RSD% < 8.7% (Table 2). This proves that the method is selective for biotin, even though other compounds also exist in the supplements.

### 3.5. Comparison of the Developed Method vs. the Reported Method in the Literature

There are several methods by which to detect biotin levels or concentrations. The classical HPLC methods were discussed in the introduction, including HPLC-UV [13], liquid chromatography-mass spectrometry (LC-MS) [2], and HPLC-FL post-column binding assays [17]. Recently, new alternatives to HPLC methods were developed for the analysis of biotin. For example, Buyuktiryaki et al. used a potentiometric sensor for biotin determination in real samples with an LOD of 0.3 × 10^−15^ M [25], and Chen et al. used a home-made surface plasmon resonance (SPR) analytical system and denaturalized bovine serum albumin (dBSA) was used to detect biotin with an LOD of 0.1 µg/mL [26].

Moreover, Donnenbreg et al. developed an improved assay for quantifying biotin based on biotin-labeled red blood cells, with a limit of quantification LOQ ranging from 1 in 274,000 to 1 in 649,000 cells [27]. Additionally, the LOD was found to be 0.03 nM in a study undertaken by Chen et al. utilizing a time-resolved fluorescence resonance energy transfer (TR-FRET) assay [28], and Buzid et al. obtained an LOD of 5 nM by using electrochemical sensing via a Nafion-modified boron-doped diamond electrode [29]. Oberbichler et al. presented a competitive binding assay using avidin and biotin-4-fluorescein (B4F) for quantification of biotin groups; the assay showed strong quenching of B4F when avidin was pre-blocked by biotinylated analyte, which overcomes the limitations of existing assays in terms of sensitivity, accuracy, and variability for different biotin derivatives [30].

Our developed CL assay of biotin showed comparable or even better sensitivity than any of the reported methods in the literature [13,14,26]. The reported chromatographic methods have many limitations. For instance, the reported HPLC-FL method used streptavidin–fluorescein isothiocyanate, which is relatively expensive and affords a very narrow linear range [14]. The LC-MS method uses expensive equipment that requires an experienced analyst to run the analysis procedure [2]. In the HPLC-UV method, detection takes place at a very low wavelength of 200 nm, which makes it very prone to interference, in addition to its relatively low sensitivity [13].

Regarding the recently reported alternative methods for biotin, the potentiometric method developed by Buyukriyaki et al. has very high sensitivity. However, it requires the use of expensive nano HRP-streptavidin and the need to regenerate the electrode after accumulation of the analyte on its surface. Moreover, the use of the SPR chip in the method developed by Chen et al. leads to reproducibility limitations due to the fact that it is not widely available in most laboratories. Furthermore, the use of flow cytometry in the method described by Donnenbre et al. is time-consuming, tedious, and limited to specific antibodies for labeling the target compound. Although TR-FRET, mentioned by Chen et al., offers a rapid and sensitive method for biotin detection, the method was not applied to real samples. It depends on fluorescence quenching induced by FRET, which is known to be subject to interference from many other factors, such as the inner filter effect. Similarly, the method developed by Buzid et al. lacks selectivity and stability and necessitates the use of complex equipment. Even the method described by Oberbichler et al. suffers from drawbacks, as biotinylated DNA with 30 nucleotides causes steric hindrances, which reduce the accuracy of biotin group determination.

Most of the methods mentioned above require expensive instruments that are not widely available and are both tedious and time-consuming to use. There are some commercially available kits or reagents for biotin detection, such as HABA reagents [24]. HABA and avidin mixture is measured at 500 nm, and when a biotinylated compound is added, it displaces HABA because of the biotin’s higher affinity to avidin, which leads to a decrease in absorption at 500 nm. In some reports, the targeted biotinylated compound has an absorbance that overlaps with the HABA assay measurement wavelength. In such cases, it is impossible to detect because of the interference encountered [22]. On the other hand, our method is simple and easily undertaken with a simple instrument (luminometer), with no need to use any extra instruments. Additionally, the proposed method is rapid, requiring only 5 min for measuring and 30 min for incubation time. A comparison of the reported methods in the literature for biotin with the developed method is summarized in Table 3.

## 4. Conclusions

In conclusion, a reliable, rapid, selective, cost-effective, and simple method was developed for the detection of biotin in supplements. Biotin was detected by the developed method, which is similar to a replacement assay with BT-NQ and free biotin in the samples, which depends on the proximity to avidin. Free biotin in the sample (supplement) binds to the avidin instead of BT-NQ, which allows the BT-NQ to be free and far away from the avidin, and thus, the CL signal is higher as the amount of free biotin in the sample increases. The detection time for biotin is only 5 min for measuring the CL, which is considered fast compared with other methods. The LOD of this developed method was 0.58 µM, and it had good recoveries from supplement samples of 91.3 to 110%. The good recovery percentage indicates that the proximity-based assay is reliable and applicable to real samples.

## Figures and Tables

**Table 1:** Accuracy and precision data for the analysis of biotin using the proposed CL competitive assay.

Studied Concentrations (µg/mL)	Accuracy (Mean% Found)	Precision (RSD, %)
Intraday		
1.00	98.8	2.05
5.00	102	0.315
20.0	99.6	4.45
100	100	1.43
Interday		
1.00	101	6.65
5.00	101	4.92
20.0	98.0	10.6
100	99.4	0.969

**Table 2:** Analysis results of biotin in its supplement tablets.

Conc. Taken (µg/mL)	Amount Found (µg/mL)	% Found ^a^	% RSD (*n* = 5)
5.00	5.52	110	8.00
20.0	18.8	94.0	4.31
40.0	36.5	91.3	8.67

^a^ Each result is an average of five separate determinations (*n* = 5).

**Table 3:** Comparison of the developed competitive CL assay of biotin with the reported methods in the literature.

Method	Sample	LOD	Linear Range	Reference
HPLC-FL	Infant formula	–	0.3–1.0 µM	[14]
LC-MS	Nutritional products	0.01–0.28 µM	0.03–9.2 µM	[2]
HPLC-UV	Biotin supplements	–	3.3–13.2 µM	[13]
Affinity-based potentiometry	Infant formula and collagen hyaluronic	0.3 FM	1–100 fM	[25]
Electrochemical sensing	Blood plasma	5 nM	–	[29]
TR-FRET assay	Solution containing biotin	0.03 nM	0.05–100 nM	[28]
SPR analytical system	Biotin derivatives	0.66 µM	0.66–6600 µM	[26]
Proximity assay	Biotin supplements	0.58 µM	1.0–100 µM	BT-NQ assay

## Data Availability

The data will be available upon reasonable request.
